# In-Vitro Activity of Nano Fluconazole and Conventional Fluconazole against Clinically Important Dermatophytes

**DOI:** 10.18502/ijph.v49i10.4701

**Published:** 2020-10

**Authors:** Najmossadat MUSAVI BAFRUI, Seyed Jamal HASHEMI HAZAVEH, Mansour BAYAT

**Affiliations:** 1. Department of Microbiology, Faculty of Veterinary Specialized Sciences, Science and Research Branch, Islamic Azad University, Tehran, Iran; 2. Department of Medical Parasitology and Mycology, School of Public Health, Tehran University of Medical Sciences, Tehran, Iran

**Keywords:** Dermatophyte, Minimum inhibitory concentration, Fluconazole, Nano-particle

## Abstract

**Background:**

Dermatophytosis is one of the most common fungal infections in humans. Antifungals such as fluconazole are effectively used for treating dermatophytosis; however, drug resistance was observed in many cases. Therefore, a newer treatment strategy is essential.

**Methods:**

This study (Conducted in the Laboratory of the School of Public Health, Tehran University of Medical Sciences, Tehran, Iran in 2018) evaluated the antifungal susceptibility of nano fluconazole compared to conventional fluconazole on dermatophyte isolates using CLSI M38-A2guidelines. Dermatophyte species isolated from clinical cases of dermatophytosis were identified using PCR sequencing techniques. Zeta potential and size of the nano particles containing fluconazole were measured; scanning electron microscope (SEM) was used to determine nano particle structure.

**Results:**

The size of liposomal fluconazole obtained was 88.9 ± 12.14 nm with –20.12 ± 3.8 mV for zeta potential. The encapsulation rate for fluconazole was 75.1± 4.2%. MIC_50_ for the three tested species was 32, 16, and 8 *μ*g/ml for *Trichophyton interdigitale*, *T. rubrum*, and *Epidermophyton floccosum* isolates, respectively. The corresponding values for nano fluconazole were 8 *μ*g/ml for the three tested species.

**Conclusion:**

MIC value for nano-fluconazole was lower than conventional fluconazole in all dermatophytes species tested; therefore, nano-fluconazole could inhibit the growth of dermatophytes better than fluconazole at a lower concentration of the drug.

## Introduction

Dermatophytosis is a common mycotic infection, affecting keratinized tissues such as skin, hair, and nails of humans and animals. Dermatophytes can be classified into three genera: *Trichophyton*, *Microsporum*, and *Epidermophyton* (1). There are about 40 species in these three genera while only 19 species are known as pathogenic. Also, based upon the affected anatomical site of the body, dermatophytosis has been classified clinically into tinea capitis (head), tinea faciei (face), tinea barbae (beard), tinea corporis (body), tinea manuum (hand), tinea cruris (groin), tinea pedis (foot), and tinea unguium (nail) (2, 3).

Although dermatophytosis is not life-threatening, it severely affects the quality of life and imposes significant treatment costs on patients (4). Dermatophytosis can easily be eradicated by topical antifungal drugs, while tinea capitis and tinea unguium do not respond well to local treatment and require systemic antifungal agents (5). Although newer oral antifungal agents used for the treatment of invasive and superficial infections have significantly improved efficacy of treatment for many fungal infections, side effects, drug interactions, and resistant organisms have considerably challenged, requiring safer and more effective treatments.

Once drug resistance occurs, it is necessary to use newer antifungal agents. Nanotechnology has been effective in many fields, particularly drug administration. Nanoparticles (NPs) are a newer generation of antimicrobial agents studied significantly in recent years (6). Nanoparticles can improve bioavailability, solubility, and permeability of drugs. Nano-liposomes have many advantages, including improved penetration and diffusion of active ingredients, selective transport of active ingredients, longer release time, and greater stability (7). Treatment with antifungal agents, particularly fluconazole (FLC), is considered as an effective treatment for some cases of dermatophytosis (8);however, there were challenges such as drug resistance and side effects. Thus, it is necessary to use newer therapeutic strategies.

In the current study, the antifungal activity of nano-fluconazole (nano-FLC) was compared to FLC on some clinically important dermatophytes.

## Materials and Methods

### Samples

Samples were taken from patients suspected of dermatophytosis, referred to the Mycology Laboratory, Tehran University of Medical Sciences, Tehran, Iran in 2018. The samples (including hair, skin, and nail) were taken, examined by direct microscopy with KOH and cultured on SDA with chloramphenicol and cycloheximide, and then incubated at 28°C for 3 weeks.

### Identification of isolates

All the dermatophyte isolates were identified based on microscopic and macroscopic properties and then subjected to molecular identification by sequencing internal transcribed spacer (ITS) regions ofribosomal DNA (rDNA) by panfungal primers ITS 1 (5’- TCC GTA GGT GAA CCT GCG G- 3’) and ITS 4 (5’-TCC TCC GCT TAT TGA TAT GC - 3’) (9).

### Preparation of fluconazole and nano-FLC

Crude powder of FLC (Sigma-Aldrich, Steinheim, Germany) was purchased from Sigma Company. The liposomal formulation of FLC was made by thin-layer film hydration (10). For producing liposomes, 5.12 mg FLC, 5 mg cholesterol, and 50 mg lecithin were used. A thin layer was formed by dissolving these two substances at a rate of 5.12 mg/ml in an organic solvent chloroform-methanolmixture (1:1) containing FLC. The final concentration of drug was 5120 μg/ml. This formulation was transferred to the laboratory for antifungal susceptibility test. The blank liposomal formulation was made without FLC.

### Evaluation of physicochemical properties of nano-FLC

Size, morphology, and zeta potential of nano-FLC were evaluated. To calculate particle size, liposomal nanoparticles produced by PCS were evaluated by using photon correlation spectroscopy. For measuring the particle in this device, formulations diluted with distilled water were transferred to the instrument and measured. Zeta potential was measured by a Zetasizer device, based on laser light scattering. Particle size distribution in the device was evaluated based on PDI (polydispersityindex). For examining the nano particle structure, scanning electron microscope (SEM) was used. A small quantity of sample was placed on a glass surface (1 × 1 cm). It was then placed inside an incubator at 37°C until the sample was completely dried. Then the particles were coated with gold; images were taken with 20000x and 40000x magnification.

### Antifungal susceptibility test

In-vitro antifungal susceptibility was tested following the guidelines of the Clinical and Laboratory Standards Institute (CLSI) M38-A2 manual (11). FLC (Sigma- Aldrich, Steinheim, Germany) and nano-FLC were diluted in standard RPMI 1640 medium (Sigma-Aldrich, St. Louis, MO, USA) buffered to pH 7.0 by 0.165 mol L-1 morpholine propane sulfonic acid buffer with L-glutamine without bicarbonate (MOPS, Sigma-Aldrich, St. Louis, MO, USA) to yield twice their concentrations; they were dispensed into 96-well microdilution trays with a final concentration of 0.25–128μg/ml for antifungal agents. Plates were stored at −70°C until testing. All isolates were cultured on Potato dextrose agar (PDA, Difco) at least for two weeks at 30°C for sporulation. Sterile normal saline (85%) was added to colonies; the suspension was collected and transferred to a tube. In order to obtain pure conidia, the suspension was filtered by a sterile filter (8μm in diameter; Whatman 40, Sao Paulo, Brazil) and collected in a sterile tube. The concentration of the resulting suspension was set spectrophotometrically (530 nm wavelength) to optical densities (ODs), ranging from 65% to 70% transmission. The inoculum suspensions diluted in RPMI-1640 were buffered (1:50) by MOPS. The final stock inoculum suspensions of each tested isolate was 1–3×10^3^ colony forming units (CFU)/ml, determined by quantitative colony counts on Sabouraud glucose agar (SGA; Difco). For the tests, 100 ml diluted cell suspension was added to each well. All tests were done in duplicate. After incubation for 60 h at 35°C, plates were read visually; MICs were determined as the lowest concentration of drug that gave approximately 80% inhibition of the growth control. The strains *T. mentagrophytes* (ATCC 4439) and *T. rubrum* (ATCC 4438) were used for quality control.

### Statistical analysis

Data was analyzed using SPSS software (ver.18, Chicago, IL, USA). MIC distributions were compared in groups for FLC and nano-FLC using Student’s t-test. Differences were statistically significant (two-tailed *P*-value≤0.05).

## Results

### Dermatophyte isolates and clinical manifestation

Out of 312 patients with suspected dermatophytosis referred to mycology laboratory of Tehran University of Medical Sciences and veterinary clinics in Tehran, 80 dermatophytes were isolated by direct examination and culture of 42 males (52.5%) and 38 females (47.5%). The prevalence of dermatophytosis was 25.6% in this study. Tinea pedis (35.0%) was the most common type of dermatophytosis. (Table 1). The distribution of all isolates is summarized in Table 2.

**Table 1:** Clinical manifestation with frequency based on age-groups and gender

***Clinical manifestation***	***Age groups (years)***						***Gender***			
	***≤10***	***10–20***	***21–30***	***31–40***	***41–50***	***>50***	***Male (%)***	***Female (%)***	***Total (%)***
Tinea pedis	-	-	4	5	8	11	17 (21.25)	11 (13.75)	28 (35.0)
Tineacorporis	1	2	5	4	3	3	7 (8.75)	11 (13.75)	18 (22.5)
Tineaungium	-	1	1	2	3	7	8 (10.0)	6 (7.5)	14 (17.5)
Tineacruris	-	1	3	2	3	4	6 (7.5)	7 (8.75)	13 (16.25)
Tinea capitis	1	2	-	-	-	-	2 (2.5)	1 (1.25)	3 (3.75)
Tinea faciei	1	-	-	-	1	-	1 (1.25)	1 (1.25)	2 (2.5)
Tinea manuum	-	-	-	-	1	1	1 (1.25)	1 (1.25)	2 (2.5)
Total	3	6	13	13	19	26	42 (52.5)	38 (47.5)	80 (100)

**Table 2:** Distribution of all isolates in various clinical manifestation

***Clinical manifestation Isolates***	***Tinea pedis (%)***	***Tinea corporis (%)***	***Tinea ungium (%)***	***Tinea cruris (%)***	***Tinea capitis (%)***	***Tinea faciei (%)***	***Tinea manuum (%)***	***Total (%)***
*T. interdigitale*	14 (17.5)	7 (8.75)	5 (6.25)	2 (2.5)	-	-	-	28 (35)
*T. rubrum*	8 (10.0)	3 (3.75)	9 (11.25)	4 (5.0)	-	-	1 (1.25)	25 (31.25)
*E. floccosum*	3 (3.75)	1 (1.25)	-	7 (8.75)	-	1 (1.25 )	-	12 (13.75)
*T. verrucosum*	2 ( 2.5 )	1 (1.25)	-	-	1 (1.25)	-	-	4 (5.0)
*M. canis*	-	4 (5.0)	-	-	-	-	-	4 (5.0)
*T. tonsurans*	-	1 (1.25)	-	-	1 (1.25)	1 (1.25)	-	3 (3.75)
*M. gypseum*	1 (1.25)	1 (1.25)	-	-	-	-	1 (1.25)	3 (3.75)
*T. schoenleinii*	-	-	-	-	1 (1.25)	-	-	1 (1.25)
Total	28 (35.0)	18 (22.5)	14 (17.5)	13 (16.25)	3 (3.75)	2 (2.5)	2 (2.5)	80 (100)

### Nano-FLC properties

**Fig. 1:**
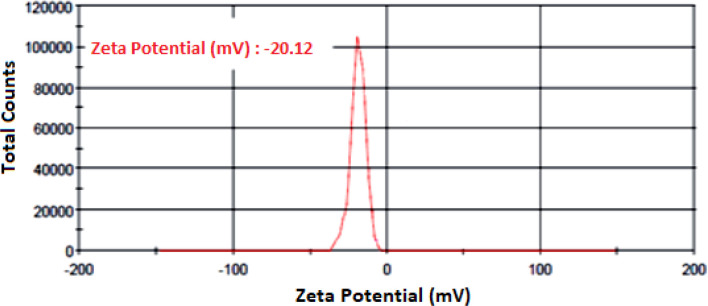
Curve of zeta potential of Nano- FLC

**Fig. 2:**
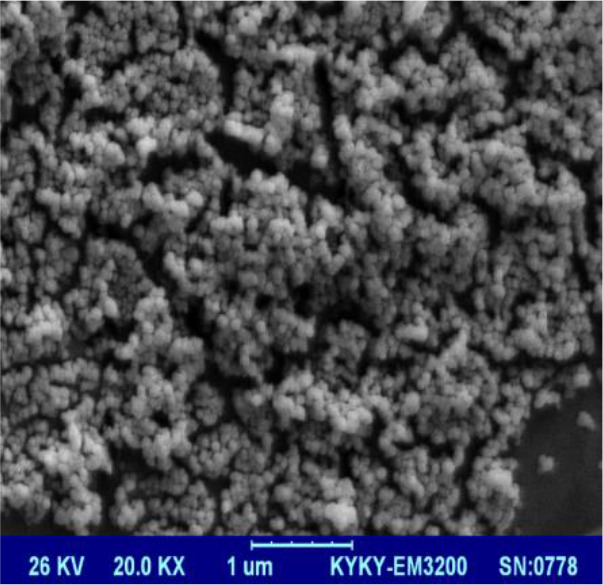
Image of nano particles with scanning electron microscope (SEM)

In this section, particle size, encapsulation rate, zeta potential, and polydispersity index of liposomal formulation of FLCare determined.Zeta potential was calculated for FLC liposomal formulation (20.12 ± 3.8 mV). Diagram of zeta potential of liposomal formulation containing FLCis shown in Fig. 1. Fig. 2 shows the result of scanning electron microscopy considering particle size distribution of nano-liposomes containing FLC. Nanoparticles are spherical (88.9 ± 12.14 nm in size). The sizes obtained from electron microscope confirmed the result of the zetasizer device. Moreover, the values obtained for PDI and Encapsulation rate were 0.448 and 75.4 ± 1.2, respectively.

### Antifungal susceptibility test

MIC_50_, MIC_90_, and MIC values of 80 clinical dermatophytes were determined. Because of the limited number of *M. canis, T. tonsurans, M. gypseum, T. verrucosum* and *T. schoenleinii* isolates, MIC_50_ and MIC_90_ were not calculated for these species. As shown in Table 3, MIC value of all dermatophytes species for nano-FLC were lower than conventional FLC. *T. schoenleinii* has the highest MIC value (128 *μ*g/ml) compared to other isolates.

**Table 3:** In-vitro antifungal susceptibilities of 80 clinical isolates of dermatophytes against FLC and nano-FLC

***Dermatophyte species***		***Antifungal agent***	***MIC^b^ range (μg/ml)***	***MIC_50_^c^ (μg/ml)***	***MIC_90_^d^ ( μg/ml)***	***P-value^e^***
*T. interdigitale* (n=28)		FLC ^a^	4–64	32	64	<0.01
		nano-FLC	1–32	8	16	
	*T. rubrum* (n=25)	FLC	4–64	16	32	<0.01
		nano-FLC	2–16	8	16	
	*E. floccosum* (n=12)	FLC	8–32	8	16	<0.01
		nano-FLC	2–16	8	16	
	*T. verrucosum* (n=4)	FLC	32–64	-	-	<0.01
		nano-FLC	8–32	-	-	
*M. canis* (n=4)		FLC	4–16	-	-	<0.01
		nano-FLC	1–8	-	-	
	*T. tonsurans* (n=3)	FLC	8–32	-	-	<0.01
		nano-FLC	8–16	-	-	
	*M. gypseum* (n=3)	FLC	16–32	-	-	<0.01
		nano-FLC	8	-	-	
	*T. schoenleinii* (n=1)	FLC	128	-	-	<0.01
		nano-FLC	64	-	-	
	Total (n=80)	FLC	1–128	16	32	<0.01
		nano-FLC	1–64	8	16	

a FLC: Fluconazol;

b MIC: Minimum inhibitory concentration;

c MIC50: minimal concentration that inhibits 50% of isolates;

d MIC90: minimal concentration that inhibits 90% of isolates;

e *P*-value: probability value.

## Discussion

In this study, the antifungal activity of nano-FLCwas studied on clinical dermatophyte isolates. As shown in Table 1, the most common cases of dermatophyte species weretinea pedis (35%) and the least was tinea manum (2.5%) and tinea faciei (2.5%). Similar to other studies in Iran, *T. interdigitale* and *T. rubrum* were the most prevalent species (3, 12–14). All dermatophyte isolates were identified using sequencing the ribosomal DNA (rDNA), internal transcribed spacer (ITS) regions. The exact identification of species is essential for epidemiological studies and appropriate treatment.

As drug resistance occurs, it seems necessary to find newer and more effective antifungal agents to overcome the resistant strains. Azoles have a low solubility in water and, therefore, their bioavailability is low. Usually, the drug is distributed unevenly in the body, and some cells take part in metabolizing the drug in the blood system. The drug is partially removed from the system before it is used. So far, some protocols have been used for the modification of antifungal agents. New drug industries have taken a step in production and administration of modern drug systems. The most important of these systems, widely used today, are hydrogels, nanofibers, nanoliposomes, niosomes, and nano-dendrimer (15).

Liposomes and nano liposomes coated with polymers have good properties, such as better adhesion to mucosal cells and better permeability than in the gastrointestinal. Research on calcitonin showed evidence of this claim (16). For the treatment of cutaneous fungal infections, permeation of the selected drug into the deeper layers of the skin is necessary (17). The small size of the lipid nano particles improves the presence of nano particles in direct contact with stratum corneum and ensures entry of the encapsulated drugs into the skin (18). In the present study, the liposomal formulation was developed and demonstrated more effective antifungal properties on dermatophyte species compared with the conventional form of fluconazole.

Particle size, zeta potential and drug-loaded was 88.9 ± 12.14 nm, 20.12 ± 3.8 mV and 75.4 ± 1.2%, respectively. Particle size and distribution width is often one of the most important quality-related parameters which affect other macroscopic properties of the nano-particle. Particles larger than 1 µm and an increase in their number can show their physical instability (19). Zeta potential is an important factor in determining the stability of the colloidal system and is the best indicator for determining the surface electric status of dispersions. In this study, the particle size of less than 1 µm and zeta potential of20.12 ± 3.8 mV indicated and confirmed the stability of the formulated nano-FLC.

To investigate the effect of new antifungal compounds, in-vitro susceptibility tests and animal models are required. In this study, antifungal susceptibility test on clinical dermatophyte isolate of standard broth microdilution method was evaluated for conventionalFLC and nano-FLC according to CLSI M38-A guidelines.MIC value for nano-FLCwas lower than FLC in all dermatophyte species tested .Lower MIC was reported for nano-FLC against *Aspergillus* species (7).Lipids can contribute to better penetration of fluconazole in the skin (20). Nano-FLC against *Candida-*resistant and sensitive species showed better anti-fungal activity than the common form of the drug (21). Many studies have shown the antimicrobial effects of FLC (13, 22, 23), but the literature lacks studies on the effects of nano-FLC against dermatophytes.

The primary significance of this study is that nano-FLC could inhibit the growth of dermatophytes better than conventional FLC. To our knowledge, this is the first study to apply nano-FLC successfully to dermatophytes.

## Conclusion

Liposomal nano particles prepared by thin-layer hydration containing fluconazole have a small and appropriate particle size and a polydispersity index of about 0.4 and a high loading percentage. Spherically and particle size uniformity of the liposomes prepared by scanning electron microscopy was confirmed. Studies on antifungal effects of this formulation *in-vitro* show that these nanoparticles have acceptable antifungal effects for the reasons discussed earlier compared to the free form of the drug.
